# Simultaneous Detection of Integrase and Antibiotic Resistance Genes within SXT Constin in *Vibrio cholerae *O1 El Tor Strains Isolated from Iran Using Multiplex-PCR

**Published:** 2012

**Authors:** Seyed Mahmoud Amin Marashi, Bahram Nasr Esfahani, Akbar Tavakoli, Sharareh Moghim, Mohammad Reza Pourshafie, Mansoor Salehi

**Affiliations:** 1 * Department of Microbiology & Immunology, Babol University of Medical Sciences, Babol, Iran*; 2 *Department of Microbiology, Isfahan University of Medical Sciences, Isfahan, Iran*; 3 *Department of Microbiology, Pasteur Institute of Iran, Tehran, Iran*; 4 *Departments of Genetics, Isfahan University of Medical Sciences, Isfahan, Iran*

**Keywords:** Antibiotic resistance pattern, Integrase, Multiplex-PCR, SXT constin, Vibrio cholerae

## Abstract

**Objective(s):**

Amongst the various antibiotic resistant elements in *Vibrio. cholerae*, SXT constin (SXT-C) is important. We were going to design a quick method for determination of antibiotic resistance gene pattern in SXT-C.

**Materials and Methods:**

Ninety four* V. cholerae *O_1_ El Tor isolates were used in this study. Antibiotic susceptibility testing, multiplex PCR amplification of SXT-C containing *dfrA1*, *sulII*, *strB* and the *int* in a multiplex PCR were done.

**Results:**

Results of our study showed that 92 (97.8%) out of 94 isolates were positive for all above genes. Multiplex PCR results correlated with the antibiotic susceptibility data obtained by using disc diffusion assay.

**Conclusion:**

Using this multiplex PCR, it would be easily possible to demonstrate the presence of antibiotic resistance in SXT-C which, in turn, allows for a suitable treatment in cholera patients causing reduction in the mortality and morbidity rate of the infected individuals.

## Introduction

Cholera is a major cause of health threat and also a major cause of death worldwide and especially in developing countries. In Iran, cholera is an endemic disease and during last decade, especially within 2004-2009 have had several outbreaks (1). 

In most cases cholera can be successfully treated with oral rehydration therapy but application of antibiotics could shorten the course of the disease. However, *Vibrio cholerae*, antibiotic resistance has been reported. Singh *et al* showed antibiotic resistance in all of strains of *V. cholerae* all over India (2). Similarly, a study in 2004 in Iran by Adabi *et al* showed resistance to trimethoprim**, **sulfamethoxazole and streptomycin (3).

In *V. cholerae*, antibiotic resistance confers by R plasmid, integrons and SXT-C (3). The SXT-C, an acronym for a conjugative, self-transmissible and integrating element is a *V. cholerae*-derived integrating conjugative element (ICE). The SXT-C (≈99 kb) encodes an integrase is a conserved sequence in the SXT-C and in its 5' end that is required for all properties of SXT-C including excision, transfer and integration. It encodes resistance to multiple antibiotics including sulfamethoxazole, trimethoprim and streptomycin. SXT-C has recently evolved as an important self-transmissible element which can integrate into the bacterial chromosome (4, 5). SXT-C has been found in several microorganisms among which *V. cholerae* O_1_ has received special attention due to continuous evolving of antibiotic resistance. This element has been reported in the members of Enterobacteriaceae as well (2, 3).

Previous studies showed the fact that, sulfamethoxazole, trimethoprim and streptomycin resistance genes were linked to each other (6). In the developing countries, cholera can be spread very fast and therefore antibiotic therapy must be applied. If cholera patients are treated quickly and properly, the mortality rate would be less than 1%. However, with untreated cholera, the mortality rate rises to 50–60% (4, 6, 7). So, the success of treatment is significantly affected by the prompt and method of treatment. Application of a rapid diagnostic assay for the identification of multi-drug resistant *V. cholerae* is also very critical. Thus, we designed a multiplex PCR for the rapid detection of antibiotic resistance genes and the SXT-C in *V. cholerae.*

## Materials and Methods

***Bacterial strains***

Ninety four* V. cholerae* O_1_ El Tor were isolated from cholera patients in different regions of Iran between 2004 and 2010. Phenotypic strain characterizations were performed using the biochemical tests. All isolates were examined following isolation on TCBS media, for motility, carbohydrate fermentation, indol, arginine dehydrolase, ornithine decarboxylase, MR-VP and growth at different NaCl concentration. All isolates were also examined for their oxidase reaction. The identities of the *V*. *cholerae* O_1_ strains were confirmed by serogrouping using growth from brain heart infusion agar (BHIA) with polyclonal O_1_ and monospecific Inaba and Ogawa antisera. *V. cholerae* strains that did not agglutinate with O_1_ antiserum were checked with O_139_ monoclonal antibody supplied (Pasteur Institute, Paris, France). *V. cholerae *O_1_ biotype El Tor strain KO194 was used as a positive control for *sulII*^+^, *dfrA1*^+^, *strB*^+^ and *int*^+^, and strain VO101 used as a negative control. 

***Antibiotic susceptibility testing***

Antibiotic susceptibility patterns to gentamicin (10 µg), doxycycline (30 µg), tetracycline (30 µg), ciprofloxacin (5 µg), oxytetracycline (30 µg), streptomycin (5 µg), ampicillin (10 µg), erythromycin (15 µg), chloramphenicol (30 µg) and co-trimoxazole (25 µg) (purchased from Becton Dickinson) were determined by the standard disk diffusion method according to CLSI guidelines (8).

***Multiplex PCR***

*V. cholerae *strains thrived overnight at 37°C in Luria–Bertani broth (Merck). Genomic DNA was isolated by DNA extraction kit (Roche, Mannheim, Germany).

**Table 1:** Sequences of primers used for detection of *int* and antibiotic resistance genes

Gene	Primer	
Integrase	*int*_forward_	5’- GCTGGATAGGTTAAGGGCGG -3’
	*int*_reverse_	5’- CTCTATGGGCACTGTCCACATTG -3’
Dihydrofolate reductase	*dfr A1*_forward_	5’-TGATGTTTACTTTCCTGAAATCCC-3’
	*dfr A1*_reverse_	5’- ATCCGTTGCTGCCACTTG -3’
Streptomycin Phosphotransferase	*strB *_forward_	5’-CGTTGCTCCTCTTCTCCATC-3’
	*strB *_reverse_	5’- TGCCTTCTGCCCTTCTCC -3’
Dihydrofolate synthase	*sulII*_ forward_	5’-TCAAGGCAGATGGCATTCC-3’
	*sulII*_reverse_	5’- ACGACGAGTTTGGCAGATG -3’

PCR amplification of target DNA was carried in a total volume of 25 µl. The reaction mixture contained 2.5 µl 10× amplification buffer [500 mM KCl, 100 mM Tris/HCl (pH 8.5), 1.0% Triton X-100], 0.5 µl 25 mM MgCl_2_, 0.3 µl each of 2.5 mM dNTPs (Fermentas, GmbH, Germany), 0.5 µl forward and reverse primers for all genes (20 ng/µl), 0.2 µl Taq DNA polymerase (5 U/µl), and 100 pg extracted DNA. PCR conditions were; initial denaturation at 94 °C for 5 min, followed by 35 cycles of 94 °C for 1 min, 60 °C for 1 min and 72 °C for 1 min, with a final extension at 72 °C for 3 min. Multiplex PCR was carried out by the simultaneous addition of primer pairs for *sulII*, *dfrA1* and *strB* in the same reaction mixture. Primer sequences used in this study are shown in Table 1. We designed these primers except primers for *int* gene that were desing by others (3). Amplified products were separated by agarose (1%) gel electrophoresis in 0.5× TBE, stained by ethidium bromide. The DNAs were extracted from the bands on the gel using gel extraction kit (Qiagen, GmbH, Germany) and then were sequenced by Macrogen Inc, Seoul, Korea.

**Table 2:** Results of analysis employing multiplex PCR to study *Vibrio cholerae* O1 strain isolated from different provinces in Iran

Sample / Control	Strains	State	No. of strains tested	Strain(s) showing presence of genes encoding:				Antibiotic resistance gene pattern*
				*sulII*	*strB*	*dfrA1*	*int*	
Sample	*V. cholerae* O_1_	Tehran	48	+	+	+	+	S, Tm , Sul
		Golestan	14	+	+	+	+	S, Tm , Sul
		Qum	9	+	+	+	+	S, Tm , Sul
		Sistan-Baluchistan	21	+	+	+	+	S, Tm , Sul
			2	-	-	-	-	---
Positive control	*V. cholerae O*_1_ KO194	---	1	+	+	+	+	S, Tm , Sul
Negative control	*V. cholerae O*_1_ VO101	---	1	-	-	-	-	---

*Sul= sulfamethoxazole; S= streptomycin; Tm= trimethoprim

## Results

Ninety four* V. cholerae *O_1_ El Tor strains were examined for the presence of antibiotic resistance genes in the SXT element. Ninety two strains showed resistance to streptomycin, trimethoprim and sulfamethoxazole by disk diffusion method. Multiplex PCR amplification using eight primers gave amplicons of different size for all strains. These results are shown in Table 2. Amplification of genomic DNA extracted from two strains of *V. cholerae* O_1_ isolated from Sistan-Baluchistan of Iran in 2004, gave negative multiplex PCR results for the *sulII*, *dfrA1*, *strB* and *int* genes*.* These strains, when tested for antibiotic susceptibility, were sensitive to all antibiotics tested for susceptibility analysis. Ninety two strains of *V. cholerae *O_1 _were positive and fragments of 157 bp *sulII*, 121 bp *dfrA1*, 430 bp *strB* and 592 bp *Int* gene were amplified by multiplex-PCR. 

## Discussion

Oral rehydration therapy is highly effective and is the principal treatment for cholera cases, but antibiotic shortens the course of the disease and reduces the severity of the symptoms. It is important to note that, this recommendation is especially valid in developed countries (5). 

Oxytetracycline is the primary antibiotic used in the cholera patients (2, 3). Trimethoprim, sulfamethoxazole and chloramphenicol have also been used for cholera treatment for which resistance has been reported in many developing countries (2, 6, 8). Initially, SXT-C was identified in *V. cholerae *O_139_ harboring trimethoprim, sulfamethoxazole, streptomycin and chloramphenicol resistance genes. This was followed by identification of the SXT element in O_1_ strains from several countries, i.e. India, Bangladesh, Mozambique and Laos between 1999 and 2004 (8-10). For the first time in world, Ramachandran *et al* showed that due to SXT element, 85% of *V. cholerae *O_1_ El Tor strains isolated in India were resistant to trimethoprim, sulfamethoxazole and streptomycin (2). Previously in our laboratory, by using disc diffusion method, Adabi *et al* identified that antibiotic resistance to sulfamethoxazole, streptomycin, and chloramphenicol were 95%, 95%, and 92%, respectively in Iran (3). In the current study, we used a quick, one step multiplex-PCR and showed the presence of antibiotic resistance in SXT-C in *V. cholerae.* Our results showed that 97.8% out of all of the *V. cholerae* El Tor strains were positive for integrase and antibiotic resistance genes*,* but 2.2% strains were negative. The results obtained in this study showed that the* V. cholerae* O_1_ El Tor strains isolated from different geographical locations were similar to the results published by Ramachandram *et al *(2) Adabi *et al *(3) in India and Iran, respectively. Furthermore, the study showed that, the SXT-C, harboring several different antibiotic resistance gene cassettes, is widely distributed among different clinical *V. cholerae* O_1_ serotypes in Iran which makes it essential for developing of a rapid detection method of antibiotic resistance pattern using molecular techniques.

## Conclusion

For first time in Iran, we can use one step multiplex PCR for detection of SXT-C and antibiotic resistance gene pattern in *V. cholerae*. This study showed that by the use of this method, physicians can choose a suitable treatment protocol for the cholera patients. Obviously, incorrect antibiotic therapy may resulted resistant strains (12). The results also show that, with the effective antibiotic therapy, we are not giving a chance to *V. cholerae* for more SXT-C transfer to other hosts. Finally, this method can be used for evaluation of the spread of antibiotic resistance in epidemiological studies. 
